# Turbulence through the Spyglass of Bilocal Kinetics

**DOI:** 10.3390/e20070539

**Published:** 2018-07-20

**Authors:** Gregor Chliamovitch, Yann Thorimbert

**Affiliations:** Department of Computer Science, University of Geneva, Route de Drize 7, 1227 Geneva, Switzerland

**Keywords:** kinetic theory, fluid dynamics, turbulence

## Abstract

In two recent papers we introduced a generalization of Boltzmann’s assumption of molecular chaos based on a criterion of maximum entropy, which allowed setting up a bilocal version of Boltzmann’s kinetic equation. The present paper aims to investigate how the essentially non-local character of turbulent flows can be addressed through this bilocal kinetic description, instead of the more standard approach through the local Euler/Navier–Stokes equation. Balance equations appropriate to this kinetic scheme are derived and closed so as to provide bilocal hydrodynamical equations at the non-viscous order. These equations essentially consist of two copies of the usual local equations, but coupled through a bilocal pressure tensor. Interestingly, our formalism automatically produces a closed transport equation for this coupling term.

## 1. Introduction

The study of turbulent flows has to face two main difficulties, namely non-linearity, which arises from the advective term in the Euler/Navier–Stokes transport equation; and non-locality, which stems from the fact that the theory of complex flows relies to a large extent [1,2] on the correlation function $Q_{ij}=\langle u_{i}^{\prime }\left(x\right)u_{j}^{\prime }\left(y\right)\rangle$—that is the average product of the fluctuating component of the velocities of fluid elements at two distant points in space. As such, $Q_{ij}$ is a fundamentally bilocal object.

These two issues are logically disjoint, and the present paper does not bring any new insight regarding the former, focusing instead exclusively on non-locality. The problem raised by bilocality is that turbulence is usually considered from the standpoint of the Navier–Stokes equation (or Euler equation in the non-viscous case), which in turn is derived from the local considerations of kinetic theory (see for instance [3,4,5,6] for a few milestones in this direction). Thus, it appears somewhat paradoxical to expect strictly local considerations to lead to a complete picture of a fundamentally bilocal phenomenon.

A different approach would be to start from kinetic theory considered from a bilocal standpoint and then on top of that build a hydrodynamics model that incorporates bilocal features from scratch. The viability of this more sensible approach crucially depends on the possibility of deriving a coherent bilocal kinetic theory of gases, which, technically speaking, amounts to obtaining a closed kinetic equation for the distribution function $f_{2}$ that describes the distribution of pairs of particles [7,8].

## 2. Two-Particle Kinetics

### 2.1. Generalized Molecular Chaos

Among the existing schemes for setting up a coherent equation for $f_{2}$, the authors and co-workers recently proposed an approach that relies on a maximum-entropy-based generalization of Boltzmann’s assumption of molecular chaos [9,10]. The key observation is that the *Stosszahlansatz*, namely the substitution $f_{2}\left(\xi_{1},\xi_{2}\right)\to f_{1}\left(\xi_{1}\right)f_{1}\left(\xi_{2}\right)$ (introducing for convenience the aggregated variable $\xi_{i}\;=\;\left(q_{i},p_{i}\right)$) before a collision, can be interpreted either as an assertion regarding the physical state of pre-colliding particles (regarding the range of validity of the *Stosszahlansatz*, see for instance [11,12]), or as a heuristic assumption which substitutes the unknown pre-collisional distribution $f_{2}$ for its least biased approximation, since the factorized distribution is precisely the distribution that maximizes entropy while being consistent with imposed marginal distributions [13] (the fact that maximum entropy distributions do not require a subjective interpretation and can be assigned an objective meaning is discussed at length in [14]).

The added value of this re-interpretation of molecular chaos is that it lends itself nicely to generalization, and in [9] it was shown how to derive a kinetic equation for the two-particle distribution. This makes it necessary to close the second-order BBGKY equation, whose collision term involves the three-particle distribution $f_{3}$. The procedure thus requires the substitution of the pre-collisional three-particle distribution with its maximum entropy approximation which is compatible with the $f_{2}$ appearing in the streaming term. The general result to keep in mind here [13] is that the maximum entropy approximation we can make on the three-particle repartition function under constraints on the bivariate marginals can be expressed as a product of bivariate functions, so that we should make

(1) $$f_{3}\left(\xi_{1},\xi_{2},\xi_{3}\right)\to G_{1}\left(\xi_{1},\xi_{2}\right)G_{2}\left(\xi_{1},\xi_{3}\right)G_{3}\left(\xi_{2},\xi_{3}\right).$$

Though elegant, this result is of limited practical scope unless one can obtain extra knowledge about the functions $G_{1,2,3}$. Fortunately, classical particle repartition functions have the peculiarity of being symmetric under exchange of particles, which implies that $G_{1}=G_{2}=G_{3}$. Hence, before collision, we are led to the ansatz

(2) $$f_{3}\left(\xi_{1},\xi_{2},\xi_{3}\right)\to G\left(\xi_{1},\xi_{2}\right)G\left(\xi_{1},\xi_{3}\right)G\left(\xi_{2},\xi_{3}\right)$$

for some function *G* which is implicitly related to $f_{2}$ through

(3) $$f_{2}\left(\xi_{1},\xi_{2}\right)=\int d\xi_{3}G\left(\xi_{1},\xi_{2}\right)G\left(\xi_{1},\xi_{3}\right)G\left(\xi_{2},\xi_{3}\right).$$

Note that compared to other closure schemes to be found in the literature, this scheme has the two-fold advantage of being constructive, and of yielding a standalone kinetic equation for $f_{2}$ and *not* a coupled system of equations for $f_{1}$ and $f_{2}$ (or possibly another function encapsulating the dependence between particles, cf. [15]).

### 2.2. Two-Particle Kinetic Equation

Once we have this ansatz at hand, the steps that usually lead to the one-particle Boltzmann equation can be replicated almost exactly in the case of the two-particle distribution. Throughout this work, we shall retain the usual assumptions of kinetic theory [7,8,16], leading us to neglect triple collisions. The streaming term for the two-particle distribution characterizing particles ‘1’ and ‘2’ will thus be altered by (1) binary collisions between ‘1’ and another particle with ‘2’ being a spectator, and (2) binary collisions between ‘2’ and another particle with ‘1’ being a spectator. Particles interact through either a hard-sphere contact interaction or a short-range, repulsive central force field [17,18].

A binary interaction is defined as occurring when two particles meet in a ball *B* of radius *R*. Defining ternary interactions is more subtle, since inasmuch as the interaction potential is the same regardless of the order of the interaction, it seems artificial to introduce a specific cutoff. We shall therefore define the range of triple collisions as the lenticular overlap of balls $B_{R}^{\left(1\right)}$ and $B_{R}^{\left(2\right)}$ characterizing the domain of interaction with ‘1’ and ‘2’, respectively. Neglecting triple collisions thus amounts to assuming that $|q_{1}-q_{2}|>2R$. Note that it is particularly important to stick tightly to the assumptions made in one-particle theory in order to guarantee that any new prediction arising in the present bilocal description can be ascribed to the statistical description considered, and not to the introduction of new physical assumptions (even though the framework presented here might eventually find its greatest relevance in systems where correlation is known to be important (e.g., granular gases [19]), in which case the assumptions made here should be relaxed and generalized).

This line of reasoning allows us to write a self-standing equation for the function $f_{2}$ describing the joint distribution of particles ‘1’ and ‘2’, which was found to be [9]

(4) $$\begin{array}{c} \left(\frac{\partial }{\partial t}+\frac{p_{1}}{m}\cdot \nabla_{x}+\frac{p_{2}}{m}\cdot \nabla_{y}\right)f_{2}\left(x,p_{1};y,p_{2};t\right)\\ \;=\int dp_{3}d\omega \frac{|p_{3}-p_{1}|}{m}\left(G_{p_{1}^{\prime },p_{2}^{\prime }}^{x,y}G_{p_{1}^{\prime },p_{3}^{\prime }}^{x,x}G_{p_{2}^{\prime },p_{3}^{\prime }}^{y,x}-G_{p_{1},p_{2}}^{x,y}G_{p_{1},p_{3}}^{x,x}G_{p_{2},p_{3}}^{y,x}\right)\\ \;\;+\int dp_{4}d\omega \frac{|p_{4}-p_{2}|}{m}\left(G_{p_{1}^{\prime },p_{2}^{\prime }}^{x,y}G_{p_{1}^{\prime },p_{4}^{\prime }}^{x,y}G_{p_{2}^{\prime },p_{4}^{\prime }}^{y,y}-G_{p_{1},p_{2}}^{x,y}G_{p_{1},p_{4}}^{x,y}G_{p_{2},p_{4}}^{y,y}\right), \end{array}$$

with $p_{1,2,3,4}$ and $p_{1,2,3,4}^{\prime }$ denoting the momenta before and after the collision, respectively. For notational convenience, we have put $q_{1}=q_{3}=x$ and $q_{2}=q_{4}=y$, as well as the shortcut $G_{p_{1},p_{2}}^{x,y}=G\left(x,p_{1};y,p_{2};t\right)$.

The first term on the r.h.s. corresponds to the contribution of the collisions possibly undergone at position x by particle ‘1’ with some particle ‘3’, while the second term accounts for the contribution of the collisions possibly undergone at position y by particle ‘2’ with some particle ‘4’. It must be emphasized that the same usual assumptions on density that allow neglecting triple collisions also imply that a binary collision occurs *either* at x *or* y, but not simultaneously at both places—this will turn out to be important when discussing the appropriate collisional invariants.

### 2.3. Collisional Invariants

Despite its un-glamorous aspect, the structure of Equation (4) is similar to the structure of the one-particle Boltzmann equation, except that the function *G* appearing in the collision integral, which comes directly from the maximum entropy formulation of the generalized *Stosszahlansatz*, is not $f_{2}$ itself but an implicit function of $f_{2}$. Our point in [10] was that although $f_{2}$ does not appear explicitly in the collision integral, this does not preclude the kind of manipulations usually performed on the Boltzmann equation, and we managed to derive appropriate collisional invariants and the bilocal equilibrium they give rise to. (Nevertheless, it seems that the standard derivation of the *H*-theorem for $f_{1}$ cannot be generalized in a straightforward way to $f_{2}$ in our formalism, even though there is no reason to believe that the two-particle entropy $H_{2}=-\int f_{2}\ln f_{2}$ does not increase over time.) The salient point in our analysis was that the formulation of local collisions in bilocal terms makes it necessary to consider a collisional invariant other than mass, momentum and kinetic energy; in particular, it happened that defining a bilocal invariant χ through the relation

(5) $$\chi \left(p_{1}^{\prime },p_{2}^{\prime }\right)+\chi \left(p_{3}^{\prime },p_{4}^{\prime }\right)=\chi \left(p_{1},p_{2}\right)+\chi \left(p_{3},p_{4}\right)$$

makes it necessary to retain $\chi_{1}=1$, $\chi_{2}=\left(p_{1}^{i}+p_{2}^{i}\right)$, $\chi_{3}=\left(p_{1}^{2}+p_{2}^{2}\right)$, but also, more interestingly,

(6) $$\chi_{4}=p_{1}^{i}p_{2}^{j}.$$

in [10] we considered only the invariant $\chi_{4}=p_{1}\cdot p_{2}$, but (6) is more general. This is due to the fact that, as mentioned above, the collision occurs at either x or y. In the former case, definition (5) with Equation (6) becomes

(7) $$\left({p^{\prime }}_{1}^{i}+{p^{\prime }}_{3}^{i}\right)p_{2}^{j}=\left(p_{1}^{i}+p_{3}^{i}\right)p_{2}^{j}$$

while in the latter it becomes

(8) $$\left({p^{\prime }}_{2}^{j}+{p^{\prime }}_{4}^{j}\right)p_{1}^{i}=\left(p_{2}^{j}+p_{4}^{j}\right)p_{1}^{i}$$

which are both trivially verified.

Armed with these four invariants, it is a simple matter to derive a bilocal equilibrium distribution describing the probability that two particles a distance *r* apart are found to have velocities $v_{1}$ and $v_{2}$. Thus we find that

(9) $$\begin{array}{c} {}^{eq}f_{2}^{\left(r\right)}\left(v_{1},v_{2}\right)\\ \;=\nu \left(\theta_{1},\theta_{2},\Psi^{\left(r\right)}\right)\exp(\alpha \left(\theta_{1},\Psi^{\left(r\right)}\right){\left(v_{1}-u_{1}\right)}^{2}+\alpha \left(\theta_{2},\Psi^{\left(r\right)}\right){\left(v_{2}-u_{2}\right)}^{2}+{\left(v_{1}-u_{1}\right)}^{T}\Psi^{\left(r\right)}\left(v_{2}-u_{2})\right), \end{array}$$

which, as might have been expected, consists of a product of Maxwellian distributions multiplied by a correlating factor. The coefficients are such that $\int dv_{1}dv_{2}{\left(v_{1}-u_{1}\right)}^{2}f_{2}=\theta_{1}$ and $\int dv_{1}dv_{2}\left(v_{1}^{i}-u_{1}^{i}\right)\left(v_{2}^{j}-u_{2}^{j}\right)f_{2}=\sqrt{\theta_{1}\theta_{2}}\varphi_{ij}^{\left(r\right)}$ (in plain words $\theta_{1}$ and $\theta_{2}$ denote the temperature at position x and y respectively, $\varphi_{ij}^{\left(r\right)}$ denotes the correlation at distance *r* of component *i* of $v_{1}-u_{1}$ and component *j* of $v_{2}-u_{2}$), and ν denotes a normalization factor.

## 3. Balance Equations

Our aim here is to work out the balance equations associated to our bilocal invariants. The very same kind of manipulations as used on the one-particle Boltzmann equation provide us with the generic expression

(10) $$\int dv_{1}dv_{1}\chi \left(v_{1},v_{2}\right)\left(\frac{\partial }{\partial t}+v_{1}\cdot \nabla_{x}+v_{2}\cdot \nabla_{y}\right)f_{2}=0.$$

Defining

(11) $$\langle A\rangle =\Omega^{-1}\int dv_{1}dv_{2}Af_{2}$$

with the bilocal density $\Omega =\int dv_{1}dv_{2}f_{2}$ allows rewriting Equation (10) as

(12) $$0=\partial_{t}\langle \Omega \chi \rangle +\nabla_{x}\cdot \langle \Omega \chi v_{1}\rangle -\langle \Omega v_{1}\cdot \nabla_{x}\chi \rangle +\nabla_{y}\cdot \langle \Omega \chi v_{2}\rangle -\langle \Omega v_{2}\cdot \nabla_{y}\chi \rangle .$$

Considering now in turn the four collisional invariants introduced above, we obtain for χ=1 that

(13) $$\partial_{t}\Omega +\nabla_{x}\cdot \langle \Omega v_{1}\rangle +\nabla_{y}\cdot \langle \Omega v_{2}\rangle =0.$$

This is a bilocal continuity equation for the bilocal density Ω(x,y), which is the exact counterpart of the standard local continuity equation.

Then, for $\chi =(v_{1}^{i}+v_{2}^{i})$, we have for the conservation of momentum

(14) $$\partial_{t}\langle \Omega \left(v_{1}^{i}+v_{2}^{i}\right)\rangle +\nabla_{x}\cdot \langle \Omega \left(v_{1}^{i}+v_{2}^{i}\right)v_{1}\rangle +\nabla_{y}\cdot \langle \Omega \left(v_{1}^{i}+v_{2}^{i}\right)v_{2}\rangle =0.$$

Using the continuity equation given by Equation (13) above, this can be rewritten as

(15) $$\begin{array}{cc} 0 & =\Omega \left(\partial_{t}+u_{1}\cdot \nabla_{x}\right)u_{1}^{i}+\Omega \left(\partial_{t}+u_{2}\cdot \nabla_{y}\right)u_{2}^{i}\\ & \;+\nabla_{x}\cdot \langle \Omega \left(v_{1}^{i}-u_{1}^{i}\right)\left(v_{1}-u_{1}\right)\rangle +\nabla_{x}\cdot \langle \Omega \left(v_{2}^{i}-u_{2}^{i}\right)\left(v_{1}-u_{1}\right)\rangle \\ & \;+\nabla_{y}\cdot \langle \Omega \left(v_{1}^{i}-u_{1}^{i}\right)\left(v_{2}-u_{2}\right)\rangle +\nabla_{y}\cdot \langle \Omega \left(v_{2}^{i}-u_{2}^{i}\right)\left(v_{2}-u_{2}\right)\rangle . \end{array}$$

We therefore obtain two copies of the pre-Euler/Navier–Stokes conservation equation for the velocity field (each acting at a different point in space), but which are coupled through a kind of bilocal pressure tensor $\langle \left(v_{1}^{i}-u_{1}^{i}\right)\left(v_{2}^{j}-u_{2}^{j}\right)\rangle$.

Next, for $\chi ={\left(v_{1}-u_{1}\right)}^{2}+{\left(v_{2}-u_{2}\right)}^{2}$ we obtain in a similar way, remembering that by definition $\langle {\left(v_{1}-u_{1}\right)}^{2}+{\left(v_{2}-u_{2}\right)}^{2}\rangle =\theta_{1}+\theta_{2}$:

(16) $$\begin{array}{cc} 0 & =\Omega \left(\partial_{t}+u_{1}\cdot \nabla_{x}\right)\theta_{1}+\Omega \left(\partial_{t}+u_{2}\cdot \nabla_{y}\right)\theta_{2}\\ & \;+\nabla_{x}\cdot \langle \Omega {\left(v_{1}-u_{1}\right)}^{2}\left(v_{1}-u_{1}\right)\rangle +\nabla_{x}\cdot \langle \Omega {\left(v_{2}-v_{2}\right)}^{2}\left(v_{1}-u_{1}\right)\rangle \\ & \;+\nabla_{y}\cdot \langle \Omega {\left(v_{1}-u_{1}\right)}^{2}\left(v_{2}-u_{2}\right)\rangle +\nabla_{y}\cdot \langle \Omega {\left(v_{2}-v_{2}\right)}^{2}\left(v_{2}-u_{2}\right)\rangle \\ & \;-2\Omega \langle \left(v_{1}-u_{1}\right)\cdot \left(v_{1}-u_{1}\right)\rangle \nabla_{x}\cdot u_{1}-2\Omega \langle \left(v_{2}-u_{2}\right)\cdot \left(v_{2}-u_{2}\right)\rangle \nabla_{y}\cdot u_{2}. \end{array}$$

Here, again, we obtain two copies of the local heat transport equation that are coupled through a bilocal heat flux.

We finally come to $\chi =\left(v_{1}^{i}-u_{1}^{i}\right)\left(v_{2}^{j}-u_{2}^{j}\right)$, for which we eventually obtain

(17) $$\begin{array}{cc} 0 & =\Omega \left(\partial_{t}+u_{1}\cdot \nabla_{x}+u_{2}\cdot \nabla_{y}\right)\langle \left(v_{1}^{i}-u_{1}^{i}\right)\left(v_{2}^{j}-u_{2}^{j}\right)\rangle \\ & \;+\nabla_{x}\cdot \langle \Omega \left(v_{1}^{i}-u_{1}^{i}\right)\left(v_{2}^{j}-u_{2}^{j}\right)\left(v_{1}-u_{1}\right)\rangle +\nabla_{y}\cdot \langle \Omega \left(v_{1}^{i}-u_{1}^{i}\right)\left(v_{2}^{j}-u_{2}^{j}\right)\left(v_{2}-u_{2}\right)\rangle \\ & \;+\Omega \langle \left(v_{1}-u_{1}\right)\left(v_{2}^{j}-u_{2}^{j}\right)\rangle \cdot \nabla_{x}u_{1}^{i}+\Omega \langle \left(v_{2}-u_{2}\right)\left(v_{1}^{i}-u_{1}^{i}\right)\rangle \cdot \nabla_{y}u_{2}^{j}, \end{array}$$

which provides a transport equation for the bilocal pressure tensor.

## 4. Non-Viscous Hydrodynamics

Our goal now is to close the balance equations, given by expressions (13), (15)–(17), by evaluating the averages over a local equilibrium solution given by Equation (9), with $\theta_{1}\to \theta_{1}\left(x\right)$, $\theta_{2}\to \theta_{2}\left(y\right)$, $u_{1}\to u_{1}\left(x\right)$, $u_{2}\to u_{2}\left(y\right)$ and Ψ→Ψ(x,y), so as to deduce the bilocal non-viscous hydrodynamical equations. (It might be argued that considering turbulent flows in the non-viscous case is somewhat vain, since viscosity plays a crucial role in the dissipation of small-scale vortices. However, the fundamental difficulty that makes the study of turbulence particularly challenging is present in the non-viscous case as well, so that from the conceptual standpoint of the present paper, considering non-viscous flows is enough for our purpose.) We have (defining at the same time the local pressure tensors $P_{1}\left(x\right)$ and $P_{2}\left(y\right)$ and their bilocal counterpart Φ(x,y)):

(18) $$\Omega \langle \left(v_{1}^{i}-u_{1}^{i}\right)\left(v_{1}^{j}-u_{1}^{j}\right)\rangle =\delta_{ij}P_{1}=\delta_{ij}\frac{\theta_{1}}{3}$$

(19) $$\Omega \langle \left(v_{1}^{i}-u_{1}^{i}\right)\left(v_{2}^{j}-u_{2}^{j}\right)\rangle =\sqrt{\theta_{1}\theta_{2}}\varphi_{ij}=\Phi^{ij}$$

(20) $$\Omega \langle {\left(v_{1}-u_{1}\right)}^{2}\left(v_{1}-u_{1}\right)\rangle =0$$

(21) $$\Omega \langle {\left(v_{2}-v_{2}\right)}^{2}\left(v_{1}-u_{1}\right)\rangle =0$$

(22) $$\Omega \langle \left(v_{1}-u_{1}\right)\cdot \left(v_{1}-u_{1}\right)\rangle =3P_{1}=\theta_{1}$$

(23) $$\Omega \langle \left(v_{1}^{i}-u_{1}^{i}\right)\left(v_{2}^{j}-u_{2}^{j}\right)\left(v_{1}-u_{1}\right)\rangle =0.$$

Hence, our conservation equations become at zeroth order, first the bilocal continuity equation (now written in components)

(24) $$\frac{\partial \Omega }{\partial t}+\frac{\partial (\Omega u_{1}^{k})}{\partial x^{k}}+\frac{\partial (\Omega u_{2}^{k})}{\partial y^{k}}=0,$$

then the bilocal Euler equation

(25) $$\begin{array}{cc} 0 & =\Omega \left(\frac{\partial }{\partial t}+u_{1}^{k}\frac{\partial }{\partial x^{k}}\right)u_{1}^{i}+\Omega \left(\frac{\partial }{\partial t}+u_{2}^{k}\frac{\partial }{\partial y^{k}}\right)u_{2}^{i}+\frac{\partial }{\partial x^{i}}P_{1}+\frac{\partial }{\partial x^{k}}\Phi^{ki}+\frac{\partial }{\partial y^{k}}\Phi^{ik}+\frac{\partial }{\partial y^{i}}P_{2}, \end{array}$$

the bilocal heat equation

(26) $$\begin{array}{cc} 0 & =\Omega \left(\frac{\partial }{\partial t}+u_{1}^{k}\frac{\partial }{\partial x^{k}}\right)\theta_{1}+\Omega \left(\frac{\partial }{\partial t}+u_{2}^{k}\frac{\partial }{\partial y^{k}}\right)\theta_{2}-\frac{2}{3}\left(\theta_{1}\frac{\partial u_{1}^{k}}{\partial x^{k}}+\theta_{2}\frac{\partial u_{2}^{k}}{\partial y^{k}}\right), \end{array}$$

and the transport equation for the bilocal pressure tensor

(27) $$\begin{array}{cc} 0 & =\Omega \left(\frac{\partial }{\partial t}+u_{1}^{k}\frac{\partial }{\partial x^{k}}+u_{2}^{k}\frac{\partial }{\partial y^{k}}\right)\Phi^{ij}+\Phi^{kj}\frac{\partial u_{1}^{i}}{\partial x^{k}}+\Phi^{ik}\frac{\partial u_{2}^{j}}{\partial y^{k}}. \end{array}$$

Finally, one might wish to obtain a transport equation for the product $u_{1}^{i}\left(x\right)u_{2}^{j}\left(y\right)$. This can be done by using Equation (25) twice to obtain

(28) $$\begin{array}{cc} 0 & =\Omega \left(\frac{\partial }{\partial t}+u_{1}^{k}\frac{\partial }{\partial x^{k}}+u_{2}^{k}\frac{\partial }{\partial y^{k}}\right)\left(u_{1}^{i}u_{2}^{j}\right)+\Omega u_{1}^{i}\left(\frac{\partial }{\partial t}+u_{1}^{k}\frac{\partial }{\partial x^{k}}\right)u_{1}^{j}+\Omega u_{2}^{j}\left(\frac{\partial }{\partial t}+u_{2}^{k}\frac{\partial }{\partial y^{k}}\right)u_{2}^{i}\\ & \;+u_{2}^{j}\frac{\partial P_{1}}{\partial x^{i}}+u_{1}^{i}\frac{\partial P_{1}}{\partial x^{j}}+u_{1}^{i}\frac{\partial P_{2}}{\partial y^{j}}+u_{2}^{j}\frac{\partial P_{2}}{\partial x^{i}}+u_{1}^{i}\frac{\partial \Phi^{kj}}{\partial x^{k}}+u_{2}^{j}\frac{\partial \Phi^{ki}}{\partial x^{k}}+u_{1}^{i}\frac{\partial \Phi^{jk}}{\partial y^{k}}+u_{2}^{j}\frac{\partial \Phi^{ik}}{\partial y^{k}}. \end{array}$$

## 5. Conclusions

It follows from our analysis that Equation (28), supplemented by expressions (25) and (27), provides a dynamical equation for the product of fluid velocities at different points in space, addressing the point raised in the introduction regarding the non-local character of complex flows. It must be emphasized that this result is deduced purely from the considerations of kinetic theory, and without resorting to any further hypotheses.

However, we considered here the full velocity field and not its fluctuating part only. Coming back to the second point regarding the non-linearity of the resulting equations, if we decompose each quantity involved as the sum of its Reynolds average plus a fluctuating component, we shall face in our bilocal Euler equation, given by Equation (25), the same problem as in the local case, with the emergence of extra stresses that are the bilocal counterparts of Reynolds stresses. Nevertheless, Equation (28) provides a dynamical equation for these stresses, so that the closure problem should not degenerate into a *hierarchical* closure problem.

It is worth reminding our assumption that the points have to be separated by a distance at least equal to the typical length characteristic of the interaction. One should therefore refrain from the temptation of taking the limit such that the points become confounded, which in the present setting would be ill-supported mathematically. That being said, this typical length is likely to be much smaller than the distances of interest in a hydrodynamical setting. It should also be recalled that the equations of hydrodynamics are notoriously robust against the breaking down of the assumptions made in first-principles derivations, so that the range of validity of the theory presented here might well turn out to be wider than expected. This will eventually be a matter for experimental confirmation or invalidation. Anyway, the theory presented here is conceived less as a fully developed scheme, and more as an invitation to explore bilocal kinetics further. We cannot but hope that we have partly reached this goal.
